# Characterization, evolution, and abiotic stress responses of leucine-rich repeat receptor-like protein kinases (LRR-RLK) in *Liriodendron chinense*

**DOI:** 10.1186/s12864-024-10560-3

**Published:** 2024-07-31

**Authors:** Zhiying Mu, Mingyue Xu, Teja Manda, Jinhui Chen, Liming Yang, Delight Hwarari

**Affiliations:** 1 State Key Laboratory of Tree Genetics and Breeding, College of Life Sciences, Nanjing Forestry University, Nanjing, 210037 China; 2 State Key Laboratory of Subtropical Silviculture, Zhejiang A&F University, Lin’an, Hangzhou, Zhejiang 311300 China

**Keywords:** *Liriodendron chinense*, *LRR-RLK* genes, Gene expression, Abiotic stress responses, Phylogeny

## Abstract

**Background:**

*Liriodendron chinense* is susceptible to extinction due to the increasing severity of abiotic stresses resulting from global climate change, consequently impacting its growth, development, and geographic distribution. However, the *L. chinense* remains pivotal in both socio-economic and ecological realms. The *LRR-RLK* (*leucine-rich repeat receptor-like protein kinase*) genes, constituting a substantial cluster of receptor-like kinases in plants, are crucial for plant growth and stress regulation and are unexplored in the *L. chinense*.

**Result:**

233 *LchiLRR-RLK* genes were discovered, unevenly distributed across 17 chromosomes and 24 contigs. Among these, 67 pairs of paralogous genes demonstrated gene linkages, facilitating the expansion of the *LchiLRR-RLK* gene family through tandem (35.82%) and segmental (64.18%) duplications. The synonymous and nonsynonymous ratios showed that the *LchiLRR-RLK* genes underwent a purifying or stabilizing selection during evolution. Investigations in the conserved domain and protein structures revealed that the LchiLRR-RLKs are highly conserved, carrying conserved protein kinase and leucine-rich repeat-like domians that promote clustering in different groups implicating gene evolutionary conservation. A deeper analysis of LchiLRR-RLK full protein sequences phylogeny showed 13 groups with a common ancestor protein. Interspecies gene collinearity showed more orthologous gene pairs between *L. chinense* and *P. trichocarpa*, suggesting various similar biological functions between the two plant species. Analysis of the functional roles of the *LchiLRR-RLK* genes using the qPCR demonstrated that they are involved in cold, heat, and salt stress regulation, especially, members of subgroups VIII, III, and Xa.

**Conclusion:**

Conclusively, the *LRR-RLK* genes are conserved in *L. chinense* and function to regulate the temperature and salt stresses, and this research provides new insights into understanding *LchiLRR-RLK* genes and their regulatory effects in abiotic stresses.

**Supplementary Information:**

The online version contains supplementary material available at 10.1186/s12864-024-10560-3.

## Background

Leucine-rich repeat-like protein kinases (LRR-RLKs) play a crucial role in plant development and stress responses [1] and encompass one of the largest receptor-like kinases in plants [2]. Their structures comprise ectodomains and cytoplasmic domains, frequently occurring in a combination of LRR and RLK domains [3]. Their biochemical structure is composed of three functional domains: an extracellular domain (ECD) that perceives signals, a transmembrane domain that acts as an anchor to the protein within the membrane, and an intracellular kinase domain (KD) that transduces signals downstream through autophosphorylation, followed by phosphorylation of exclusive substrates, subsequentially [4]. In addition, the LRR-RLK ECD is characterized by varying numbers of LRR repeats that facilitate sensing several ligands, including small molecules, peptides, or entire proteins [5]. On the other hand, the LRR-RLK KD is common in protein kinases, constituting 12 conserved subdomains that exhibit an identical three-dimensional catalytic primary two-lobed structure; known for their vital functions in enzymatic roles [6, 7].

Furthermore, a typical plant LRR-RLK family is classified into 15–29 groups and subgroups based on the phylogenetic analysis of the KDs and denoted based on the subgroup classification of *A. thaliana* LRR-RLKs, numbered in Roman numerals [8]. The classification of the LRR-RLKs largely depends on the phylogeny of KDs due to the ambiguity in other conserved domains [9]. Nonetheless, the LRRs share a common structure defined by a 20–28 residue expanse rich in leucine, and seven discrete sub-groups have been identified sharing a conserved LxxLxLxxNxL(s/t)GxLPxxLxx (where L denotes the hydrophobic amino acid, and N stands for asparagine, threonine, serine or cysteine, and x is a variable residue) [10]. In addition, the highly conserved region ‘LxxLxLxxN’ in LRRs conforms to a curved parallel β-sheet lining in the inner circumference of their solenoid structure, while the conserved ‘L(s/t)GxLP’ region forms the plant-specific β-strand which affects the positioning of the LRR stacks into a superhelical assemblage [11].

Extensive research has characterized the *LRR-RLK* gene family in several plants and showed their responses in the regulation of a wide range of biological processes in plants, such as growth and development, microsporogenesis and embryogenesis, plant immune response against pathogens, and tolerances to various abiotic stresses like heat, cold, drought, salt, and nutrient treatment [12]. For instance, an LRR-RLK protein HSL3 has been shown to negatively regulate the stomatal closure by modulating the level of H_2_O_2_ in guard cells, thereby the HSL3 was concluded to participate in the regulation response of drought stress [13]. Overexpressed novel cold tolerance *LRR-RLK* gene (*MtCTLK1-OE*) in *M. truncatula* increased cold tolerance through activating the C-repeat-Binding Factor (CBF)-pathway, antioxidant defense system, and proline accumulation [14]. A phytosulfokine receptor (PSKR) in rice upregulated by ABA increased stomatal closure, regulated the ROS activity in the guard cells, thereby enhancing drought tolerance in Arabidopsis. Further research has demonstrated that the *OsPSRR15* enhances drought stress tolerance through direct interaction with *AtPYL9* and its orthologue *OsPYL11* through its kinase domain in the plasma membrane and nucleus [15]. In other studies, *LRR-RLK*s have been proven to perform a dual function in heat tolerance and biotic stress resistance to *P. strigiform* F. *sp. triitcii* by interacting with the *TaDJA7* and activating the *HSP*s. In wheat, the somatic embryogenesis receptor kinase (*TaSERK1*) showed increased resistance to stripe rust disease caused *by Puccinia striiformis* f. *tritici* and showed upregulated expression levels under high temperatures. Additional analysis demonstrated that this upregulation results from the exogenous application of salicylic acid and brassinosteroids. Thus, conclusions were made that *TaSERK1* probably interacts with the phosphorylated *TaDJA7*, a heat shock protein 40 subfamily, under relatively high temperatures mediated by the salicylic acid and brassinosteroids signal pathways to increase heat resistance [16]. Previous research has also shown an *LRR-RLK* gene, *Phloem Intercalated with Xylem-Like 1* (*PXL1*) in Arabidopsis to be induced by cold and heat stress, and phosphorylate *AtHIRD1* and *AtLHCA1* in the regulation of the signal transduction pathways under temperature fluctuations [17].

The studies mentioned above provide substantial evidence of the involvement of the *LRR-RLK* genes in regulating various environmental stresses and their active participation during plant growth and development. Additionally, several publications have identified the *LRR-RLK* genes in Arabidopsis [18], *Zea mays* [19], rosacea plants [20], Saccharum [21], and Gossypium species [22]. However, the LRR-RLKs have not yet been identified and their abiotic stress response elucidated in *Liriodendron chinense* (Lchi). The Liriodendron genus comprises two prominent species, the *Liriodendron chinense* and *Liriodendron tulipifera* [23]. The species vary from annual plants due to their woody secondary growth and perennial habit [24].

Similarly, several abiotic stresses affect the *Liriodendron chinense* growth and distribution, including cold, heat, drought, light, and nutrient utilization [25]. To gain insight into the response of *LRR-RLK* genes in *L. chinense*, we investigated their biochemical properties and expression patterns to various abiotic stresses through the qPCR expression analysis. These analyses provide a firm foundation for further biological experimentation.

## Methods

### Identification and classification of *LRR-RLK* genes

The genomic and protein sequences of *Liriodendron chinense* were obtained from the TreeGene database (https://treegenesdb.org/org/Liriodendron-chinense; accessed on 30 March 2023); those of *Arabidopsis thaliana* were retrieved from the from TAIR (https://www.arabidopsis.org/browse/genefamily/leuc.jsp; accessed on the 30th of March 2023) and used as a reference in the identification process. Other *LRR-RLK* genes from different plants were obtained from Phytozome v13 (https://phytozome-next.jgi.doe.gov/). To identify *LRR-RLK* genes in the *Liriodendron chinense*; putative PKs were initially obtained by searching the Hidden Markov Models of the typical Pkinase clade [Pkinase (PF00069) and Pkinase_Tyr (PF07714)] obtained from the Pfam database v.28 [26], against the proteome of *L. chinense* using the simple HMMER search in TBtools [27]; with an E-value cut-off of 0.0001[20]. Typical PKs were identified with coverage of the Pfam domain model of at least 50%, after the screening, and CDD search from both the NCBI CDD (https://ncbi-nlm-nih-gov.brum.beds.ac.uk/Structure/bwrpsb/bwrpsb.cgi); and SMART (http://smart.embl-heidelberg.de/) was used to authenticate the identified putative LchiLRR-RLKs further. In addition, previously defined HMMs of different typical PK groups and subgroups (https://github.com/lileiting/Plant_Pkinase_fam.hmm) [28], were used to classify the identified PKs and subgroups at an E-value cut-off of 0.0001. The HMM subfamily was classified based on the phylogenetic classification of model plant species, *A. thaliana, O. sativa,* and *P. patens* [28].

### Multiple alignments and phylogenetic analysis

Multiple sequence alignments were performed on the full-length amino acid sequences of LRR-RLK proteins in *L. chinense, A. thaliana, O sativa*, and *P. patens* with the MUSCLE program using default parameters as implemented in Geneious Prime v. 2024.0 [29]. Subsequently, phylogenetic trees were constructed based on the protein multiple sequence alignments (MSA) using the neighbor-joining tree (NJT) method and the Jukes-Cantor genetic distance model, the bootstrap test value was set at 1000 times. To confirm the result from the NJT method, another LRR-RLK phylogenetic tree was constructed using the UPGMA method in Geneious Prime v. 2024.0 with a similar bootstrap value and other parameters held constant. All identified LRR-RLK proteins were predicted for subcellular localization using the DeepLoc-2.0 tool (https://services.healthtech.dtu.dk/services/DeepLoc-2.0/; accessed on the 27th of April 2023).

### Gene structure and conserved motif analysis

The amino acid properties of the LchiLRR-RLKs were investigated by the MEME online tool (http://meme-suite.org/; accessed on 23 April 2023) using the following parameters; optimum width, 5–60; several repetitions, maximum number of motifs, 15 (Fig S1), to identify conserved motifs [30]. To confirm the conserved motifs in the LchiLRR-RLKs, the InterPro software was used (http://www.ebi.Ac.uk/InterPro/; accessed on 23 April 2023). In addition, the conserved domain (CDD), motif number and arrangements, and cis-elements were analyzed per each group. Individual LRR-RLKs were assessed for number, arrangement, and types present for the CDD and Motif analyses. To display the *LchiLRR-RLK* gene structures, the *L. chinense* chromosome gff. file obtained from the Hardwood Genome Database (https://hardwoodgenomics.org, accessed on 25 April 2023) was used in the TBtools software, and exon–intron arrangements of each *LchiLRR-RLK* were generated [27]. Similarly, the *LchiLRR-RLK* gene exon–intron arrangements were analyzed per each group.

### *Cis*-Acting element analysis

The nucleotide sequences of the *LchiLRR-RLK* gene family obtained from the Hardwood Genome Database (https://hardwoodgenomics.org, accessed on 25 April 2023) were used for the regulatory cis-elements information. The upstream 1500 bp from the region corresponding to the start codon was regarded as the promoter sequence region, then all the putative cis-elements were obtained by the Plant Care Online software (https://bioinformatics.psb.ugent.be/webtools/plantscare/html; accessed on 26 April 2023). The obtained putative cis-elements were categorized into: plant growth and development, plant hormone responses, and abiotic and biotic stress response. Similarly, the cis-elements were analyzed per each group and results were summarized in a heatmap using the TBtools software.

### Chromosome location, gene duplication, and synteny analysis

The chromosome locations of each *LRR-RLK* gene were obtained from their genome resources. Then the TBtools software was used to map the gene on the corresponding chromosome. For synteny analysis, genome regions that showed syntenic relationships were identified using the McScanX in Tbtools with default parameters. The synonymous and non-synonymous ratios (Ka/Ks) were calculated using the Ka/Ks calculator in Tbtools. The tandem and segmental repeated genes were searched by comparing the *LRR-RLK* gene in their corresponding positions in chromosome/scaffolds, and adjacent genes were designated as tandem duplicated genes.

### Plant material treatment, RNA isolation, cDNA synthesis, and the RT-qPCR

Somatic embryo-fetal regenerated seedlings of *L. chinense* grown in an incubator under white light (16 h light and 8 h dark) with constant growth and vigor were selected at 7 weeks for qRT-PCR. The selected plants were divided into three batches of fifteen plants per batch and three biological replicates were set for each stress treatment. The seedlings were transferred into three separate incubators: 4 ^O^C for cold stress treatment, 35–40 ^O^C for heat stress treatment, and drought conditions were set at 40% polyethylene glycol/PEG6000. Both the tender and mature leaves of the seedlings were extracted at 0 h (0 h; set as control), 3 h, 24 h, and 3 days after stress treatment then placed in liquid nitrogen (N) for quick freeze and stored at -80 ^O^C for additional experimentation.

Total RNA was extracted from the tender leaves using the HiScript® III 1st Strand cDNA Synthesis Kit (+ gDNA wiper) (Nanjing Vazyme Biotech Co., Ltd; China) according to the manufacturer’s instructions. RNA degradation and contamination were monitored on 1% agarose gel, and concentration was measured using the Nanophotometer spectrophotometer (IMPLEN, CA, USA). Samples of total RNA (1 μg) were used to synthesize cDNA; the cDNA was subsequently diluted to 100 ng/μL and used as a template for RT-qPCR analysis.

To determine the expression patterns of the *LchiLRR-RLK* genes under three abiotic stresses (cold, heat, and salt), 20 *LchiLRR-RLK* genes were selected based on the cis-elements result. The SYBR-green in the Roche LightCycler®480 real-time PCR system (Sweden) was used, and all qRT-PCR primers were designed by Primer 5.0 and are listed in Table S1. The technical replicates were set as three per treatment and gene expression values were averaged. Actin was used as the reference gene and 18 s rRNA was used as the internal reference. The relative gene expression levels were determined using the 2^−ΔΔCt^ method [31].

## Results

### Genome-wide identification and classification of the LRR-RLK genes in *Liriodendronchinense*

**Fig. 1:**
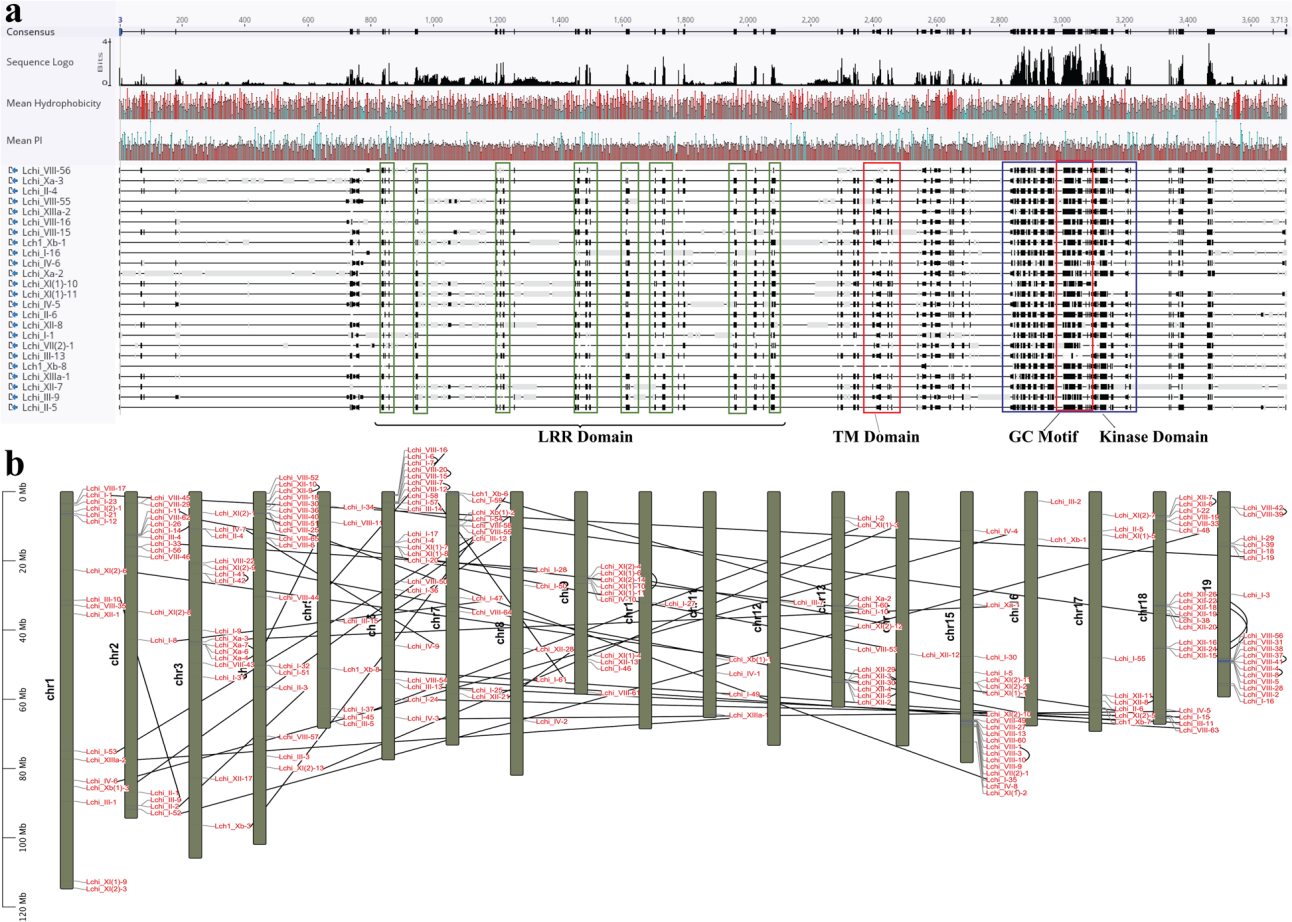
LRR-RLK gene characterization in *L. chinense*. **a** shows the multiple sequence alignments (MSA) of the LchiLRR-RLK representative protein sequence in each group as generated by the Geneious Prime software, indicating the conserved motifs and domains labeled in black below. The label LRR domain shows the Leucine-rich repeats while the TM shows the transmembrane domain conserved in all the representative sequences, the GC motif is also marked in yellow boundary within the Kinase domain. In addition, the Isoelectric point (pI) [ranging from 4.77 to 10.41] of each sequence are shown as graphs in the upper part of the figure (marked in red). **b** shows the gene location and collinearity of the *LchiLRR-RLK* genes located on 17 chromosomes, each chromosome is colored in dark green, gene labels, and positions are denoted in red while the gene collinearity is shown with linking black lines

We searched the annotated genes in the *Liriodendron chinense* genome resource for putative PKs and identified 1488 typical PKs (Table S2). After removing redundant, overlapping, and sequences lacking the LRR-RLK conserved domains, 233 LchiLRR-RLK protein sequences remained (Table S3). The obtained LchiLRR-RLK protein sequences carried an extracellular domain (ECD), a transmembrane domain, and an intracellular kinase domain (KD) (Fig. 1a). In addition, the LchiLRR-RLK ECD was branded by varying numbers of LRR. The obtained PKs were then classified and renamed into groups and subgroups based on the previous classifications in Arabidopsis and rice model plants (Table S3) [4]. LchiLRR-RLK protein classification showed 15 groups, named in Roman numerals (I-XV) following previous publications [4]. Groups VII and XI were the largest carrying 76 and 48 members, respectively. The other groups did not have more than 25 members, of which group IV had the least members, with only 1 sequence. Furthermore, the protein sequence lengths varied, ranging between 124 and 1454, and the isoelectric point ranged from 4.77 to 10.41, suggesting that the obtained LchiLRR-RLKs ranged from weakly acid to strong basic (Table S3); the average molecular weight of the identified LchiLRR-RLKs was 88.451 kDa and all the proteins showed a cellular localization in the plasma membrane (Table S3).

### Gene chromosomal location, duplications, and collinearity

To obtain further insights on the gene locations of the identified LchiLRR-RLK protein sequences, the TBtools software was used to map each gene location on the chromosome and contig (Fig. 1b; Table S3). Results showed that the *LchiLRR-RLK*s were unevenly distributed on 17 chromosomes and 24 contigs, each chromosome carried at least 12 and at most 27 *LchiLRR-RLK* genes. *LchiLRR-RLK* gene chromosome positions are shown in Table S3. Additionally, analysis of the obtained genes' collinearity exhibited 67 paralogous gene pairs, constituting almost half the total (Fig. 1b; Table S4). This finding further suggested gene duplication events and gene family expansion within the *LchiLRR-RLK* gene family. Therefore, to understand the mode of gene expansion, we compared two main gene duplication events: tandem and segmental duplication events [32, 33] (Fig. 1b; Table S4). Results showed that of the total 67 duplicated gene pairs, 24 pairs were tandem arrays, contributing 35.82% (48/134) of the duplicated genes. In addition, most of the tandem duplications were obtained in groups VIII and XII contributing 67% (16/24) of the total tandem arrays. On the other hand, 43 gene pairs were by segmental duplications and contributed 64.18% (86/136) to the *LRR-RLK* gene family expansion in *L. chinense* (Table S4). Most of the segmental duplications were obtained in gropus I and XI contributing 23% (20/86) and 12% (10/86) of the total number, similar results were obtained in Rosaceae plant genomes [20].

The synonymous and nonsynonymous values and their ratios are used to estimate the selection pressure of a given protein or DNA experience. To determine the source of duplicate genes, we calculated the synonymous and nonsynonymous values and their ratios (Table S4). A total of 138 linked genes were obtained, of which all the genes investigated for substitution mutation had a Ka/Ks ratio less than 1 (ka/ks < 1), signifying a purifying or stabilizing selection of the *LchiLRR-RLK* genes during the evolutionary process.

### Motif, gene structure, and domain conservation analyses reveal conserved evolution

**Fig. 2:**
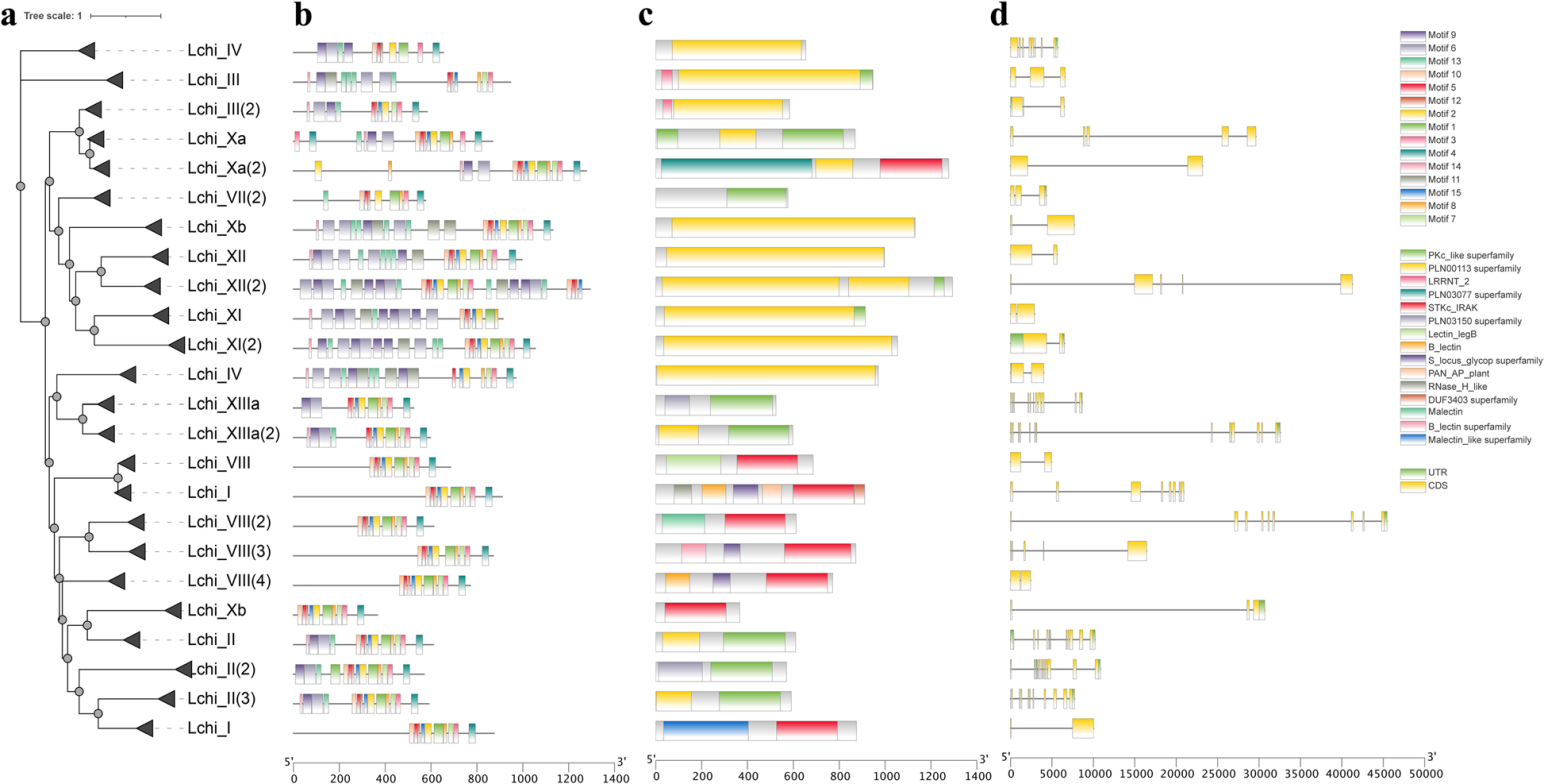
LchiLRR-RLK protein motif structure and arrangement. **a** The Phylogenetic relationship of LchiLRR-RLK representative proteins, generated by the TBtools software and beautified using the ITOL online tool. **b** shows the motif arrangements in representative LchiLRR-RLK proteins as analyzed by the MEME online tool; the motifs detected were numbered 1–15 (Fig S1) shown in the key top right corner. **c** shows the conserved domain arrangements in different colors fully described in the key bottom right corner. **d** The exon–intron number and arrangements of representative LchiLRR-RLK proteins. The exons are depicted in yellow, while the introns are shown in green. The scales below indicate the approximated lengths

To gain insight into the 233 LchiLRR-RLK protein functions, we computed the conserved motif numbers and arrangement (Fig. 2; Table S5). The investigated proteins clustered based on their similar motif arrangements and possible phylogenetic relationships (Fig. 2a). In detail, most groups had 9 motifs present in each protein except for groups III and XII which had 10 to 15 motifs (Fig. 2b). In addition, we noticed that motifs 9 and 10 among others were to a greater extent present in the groups III, XI, and XII only; motifs 5 and 2 were abundant in all groups, suggesting their full conservation.

Generally, the basic structure of the LRR-RLK gene comprises a PK domain and an LRR domain. We investigated the conserved domain in identified gene candidates. In this study, we showed that different groups have different compositions of protein domains (Fig. 2c; Fig S2; Table S5). However, most subgroups carried a Pkinase domain and an LRRNT_2 (leucine-rich repeat N-terminal) (Fig. 2b; Table S5; Fig S2). We also observed that some groups like the XI, II, and XIII had a mixture of the Pkinase and the Pkinase_Tyr. Group XI had the most conserved domains, carrying 6 conserved domains. Gene structure prediction is vital in comprehending the gene evolution of a gene family [25]. In this study, we analyzed the gene structures of 233 *LchiLRR-RLK* genes (Fig. 2d; Fig S2). Results showed that exon and intron numbers varied with gene sequences. Groups Xb, Xa-1, VII-2, XII, XI-1, III, IX, and Xb-2 had exon ranges between 1–3, accompanied by 2 or 1 introns flanking the ends. The rest of the gene groups had more than 3 exons accompanied by at least 2 introns. Specifically, group XIIIb had the greatest number of exons. Interestingly, groups with fewer exon numbers carried elongated exons, and their intron structures were smaller and almost of similar sizes depending on the groups, except for the groups Xb-3 and XI[2] which were flanked by elongated introns at ends.

### Phylogenetics of the LRR-RLK gene family

**Fig. 3:**
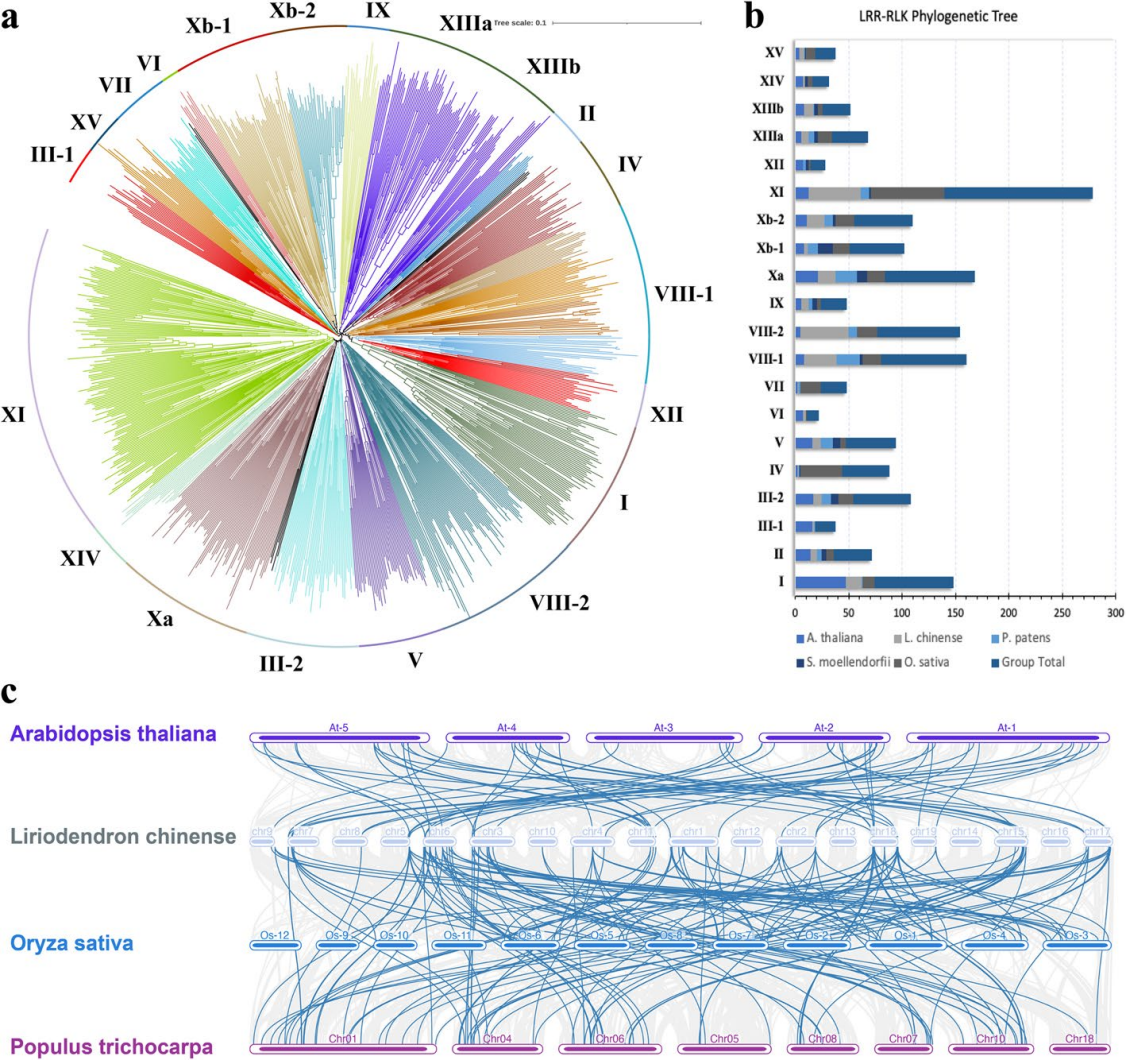
*LchiLRR-RLK* gene evolution. **a** The Phylogenetic analysis of LchiLRR-RLK shows the evolution of 1032 LRR-RLK full protein sequences. The phylogenetic tree was generated using the neighbor-joining tree (NJT) method in Genious Prime software; prior, sequences were merged and aligned using MUSCLE. Different color backgrounds show different subgroups denoted as I-XV. Furthermore, LchiLRR-RLK proteins were categorized into subgroups based on their clustering, shown with different color branches and boundaries. **b** The summary of the total number of LRR-RLK proteins present in each plant and group in Fig. 3a. The bar graph shows varying group sizes of LRR-RLKs in each plant analyzed. **c** Shows interspecies *LRR-RLK* gene collinearity between *L. chinense*, *A. thaliana*, *O. sativa*, and *P. trichocarpa*. Blue curvy lines show collinear *LRR-RLK* genes between the four plant species, each chromosome was named above

Systematic classification of a gene family based on the protein phylogeny facilitates the building of functional and genomic studies. In this study, 932 LRR-RLK full protein sequences from five plant species, *L. chinense, A. thaliana, O. sativa*, S*. moellendorfii,* and *P. patens*, were used to construct a phylogenetic tree using the neighbor-joining tree (NJT) and the Maximum-Likelihood methods (Fig. 3a; Fig S3). Results displayed a clustering of protein sequences into various groups and subgroups consistent with research in Populus [34], Gossypium species [22], and others. We observed 20 cluster groups that had diverged from 3 main branches, forming 15 groups and 5 subgroups. Nonetheless, all the cluster groups were observed to have diverged from a common ancestral protein. A deeper analysis showed that one of the main branches carried most of the LRR-RLK groups, 11 in total, while the remaining had 1 and 6 groups. This fact suggests that the LRR-RLKs evolved mainly from a single ancestral protein that diversified possibly through speciation adaptation and other evolutionary measures. Comparisons of the LRR-RLK protein members in various cluster groups showed that groups VIII and XI had the most protein numbers, 104 and 117, respectively (Fig. 3b).

In contrast, subgroups VI and XII had the least number of protein sequences 11 and 143, respectively. Additionally, the *L. chinense* LRR-RLKs were fully presented in all evolutionary groups, suggesting that the LRR-RLKs have been fully conserved in the *L. chinense* or rather the LchiLRR-RLKs have undergone a series of evolution and duplication, thereby generating new LRR-RLK protein. In total, this finding suggests differences in the LchiLRR-RLK protein conservation, thus increasing their functionality.

### Plant synteny

Gene collinearity within different plant species genes may also reflect phylogenetic relations and possibly similar gene functions. This research used the TBtools software to compute *LRR-RLK* gene collinearity between four plant species: *A. thaliana, L. chinense, O. sativa*, *and P. trichocarpa* (Fig. 3c). Results showed a dense linkage of several genes. Specifically, *L. chinense* had 55, 48, and 89 orthologous gene pairs with *A. thaliana, O. sativa*, and *P. trichocarpa*, respectively, suggesting a closer evolutionary relationship between *L. chinense* and *P. trichocarpa* than any other plant.

### *Cis*-regulatory elements

**Fig. 4:**
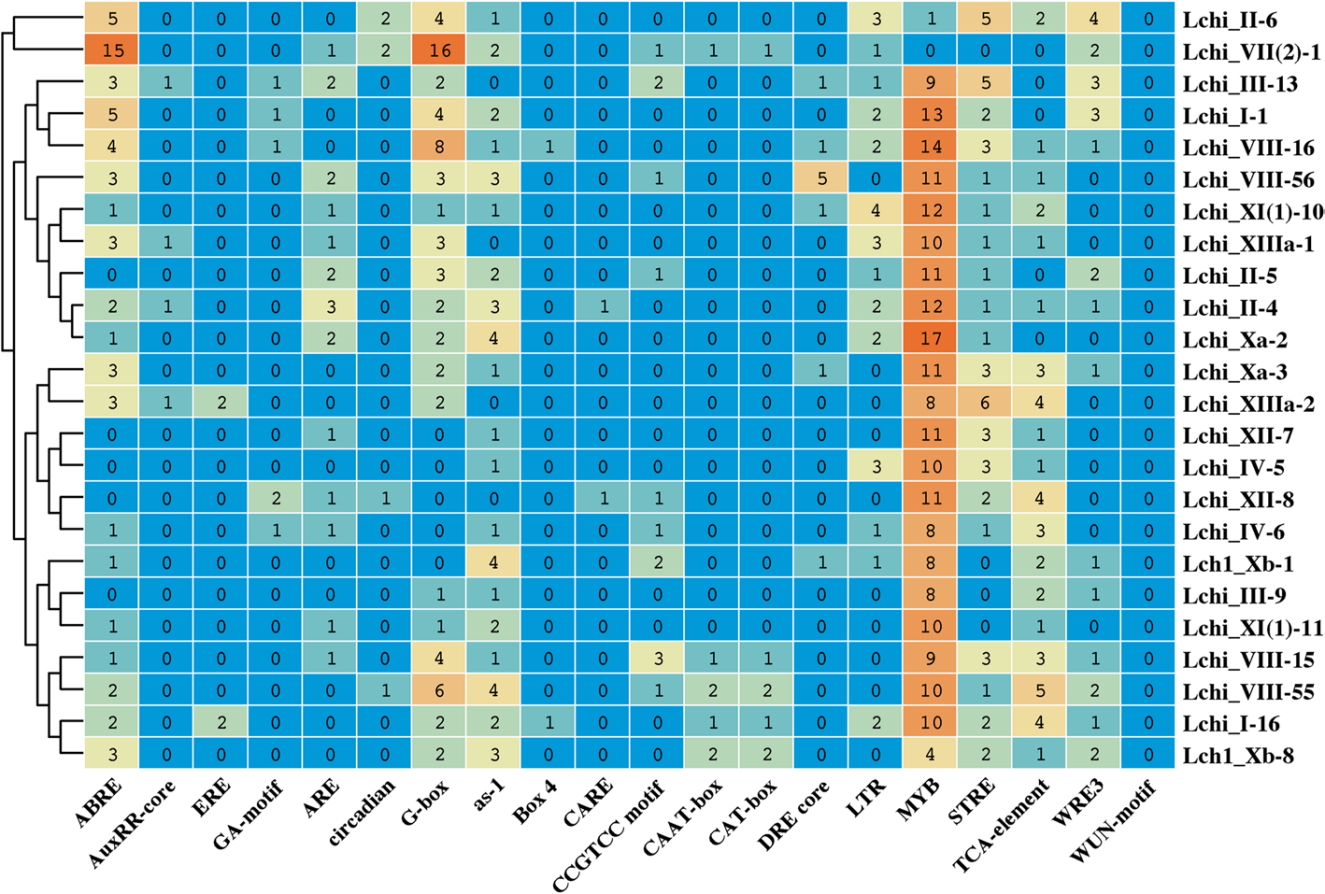
Cis-regulatory element analysis. The total number of putative cis-elements in the promoter regions of 24 representative *L. chinense LRR-RLK* genes. Numbers in the boxes represent the total cis-regulatory elements and different colors show the ranges of cis-elements

Evaluation of the cis-regulatory elements in the promoter region is critical in understanding transcriptional regulation and gene function [35]. We searched for the optimum promoter region. protein sequence alignments of the identified *LchiLRR-RLK* genes [36]. We considered the 1.5 kb region a potential promoter region containing potential regulatory elements that influence gene expression [37]. 5056 putative elements were identified and categorized into three response factors, growth and development, plant phytohormone, and biotic and abiotic responses using the Plant Care Online Database (Fig S3; Table S6). However, 24 representatives are shown in the manuscript for presentation purposes (Fig. 4). The cis-element abundancies were not consistent in all the 233 *LchiLRR-RLK*s.

Nonetheless, we noted that distributions of the cis-elements followed a similar pattern within identical groups and subgroups. Comparisons in the response factors mentioned above showed an overrepresentation of the cis-elements in the biotic and abiotic responses constituting 55.22% of the total identified cis-elements, suggesting that the *LchiLRR-RLK* genes are more invested in biotic and abiotic stress functional roles. Furthermore, the phytohormonal and growth and development responses constituted 16.8% and 28%, respectively. In-depth analysis showed that the *LchiLRR-RLK* groups VIII and I had the most cis-element in all the response factors analyzed probably due to the fact they have many members present compared to other groups. Particularly, this research focused more on the abiotic stresses; therefore we considered several cis-regulatory elements involved in the abiotic stress responses including the DRE-core, LTR, STRE, MYB., etc. Specifically, the MYB cis-elements were present in all the *LchiLRR-RLK* genes. At the same time, the STRE and LTR elements were also present in almost all the *LchiLRR-RLK*s, suggesting that *LRR-RLK*s in *L. chinense* respond to abiotic stresses including temperature and drought.

### Protein interaction and protein structure

**Fig. 5:**
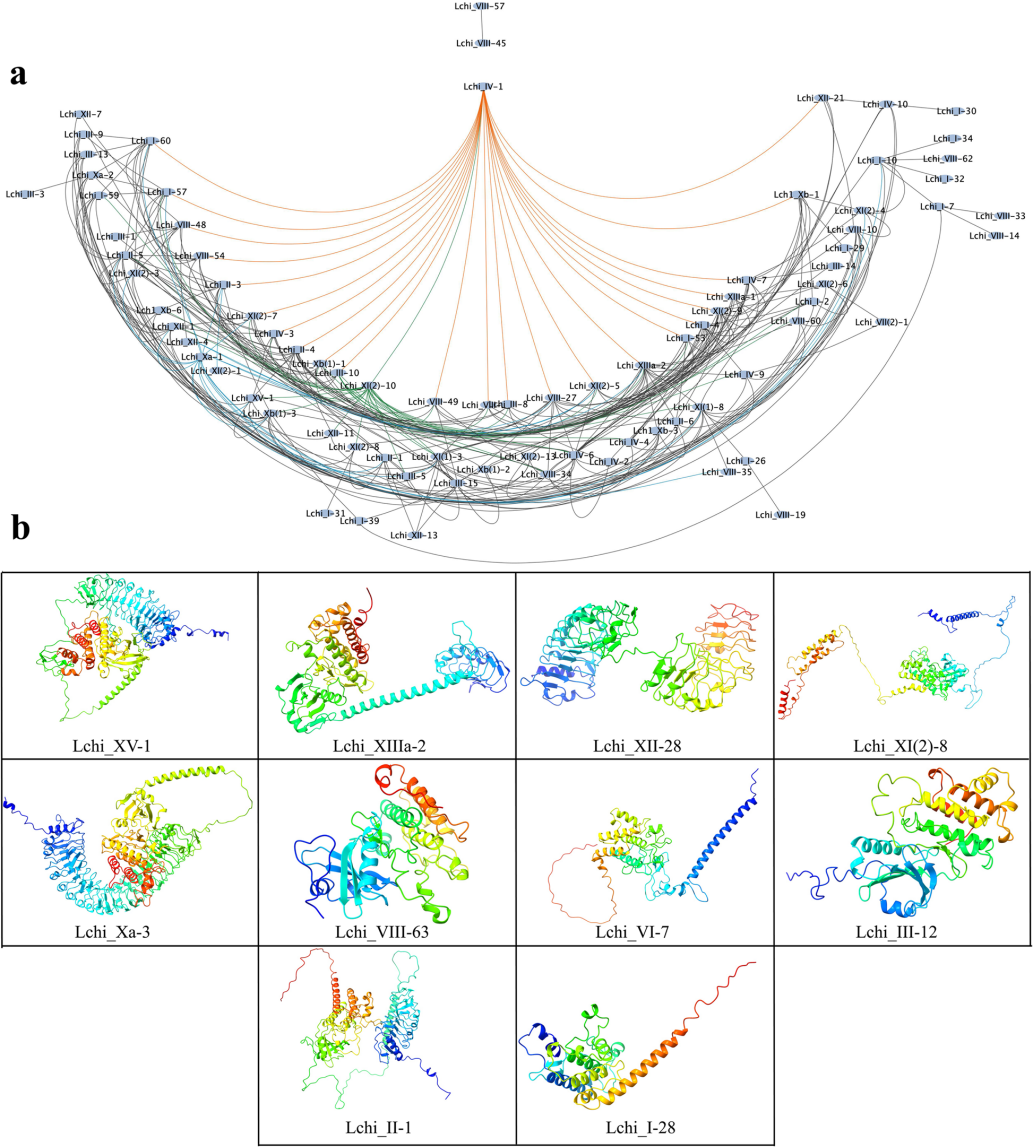
Protein interaction and structure analysis. **a** Protein–protein interaction network for LRR-RLKs analyzed using the STRING website (http://string-db.org, accessed on 25 June 2023) using the full-length protein sequences of the LRR-RLK family. *Arabidopsis thaliana* was used as a reference plant species. Each LRR-RLK protein is labeled at the node, and the lines depict interactions. **b** The 3D protein structure prediction of 10 LRR-RLK proteins, showing the potential strands, helices, and coil formation

Protein–protein interaction (PPI) analysis is crucial in elucidating protein function and the impact of protein absence or presence. This study used the Online String database to investigate the protein interaction between various LRR-RLK proteins (Fig. 5a). We observed that the LRR-RLK protein groups were densely interconnected. Individual LRR-RLK protein groups interacted with multiple groups probably for efficient biological functions. Indicating that the LRR-RLK gene groups in *L. chinense* interact for full protein function. In detail, most proteins were linked with the Lchi_IV-1 of group IV showing a possibility that Lchi_IV-1 acts as a control hub mediating several protein functions.

Previously, Chen et al. [11] have shown that plants with numerous continuous LRRs and few insertion segments in the ectodomain tend to stack into super helical shapes for sensing various ligands in signal activations [11]. To gain insight into the protein structures of LchiLRR-RLK proteins, we searched for the homology models using the SWISS-model online tool (Fig. 5b). Previous research has established that LRR assembly structures are predictable due to the high conservation of the LRR repeats, with the “LxxLxLxxN” forming the inner side of the superhelix, while the “xLs/tG” form the plant-specific second β-sheet on the lateral side, and the remainder forming the backside [19]. In this study, ten representative LchiLRR-RLK proteins showed different protein structures however, those from groups XI, XII, XIII, and XV exhibited similar protein structures. Generally, the LchiLRR-RLKs had numerous LRRs that formed the superhelices and buried their hydrophobic patches inside (Fig. 5). Additionally, the conserved residues of the LRR backbone were more hydrophobic than the variable residues, nonetheless, the variable residues had lower hydrophilicity than we predicted to aid in proper protein folding.

### Responses of *LchiLRR-RLK* genes to abiotic stresses

**Fig. 6:**
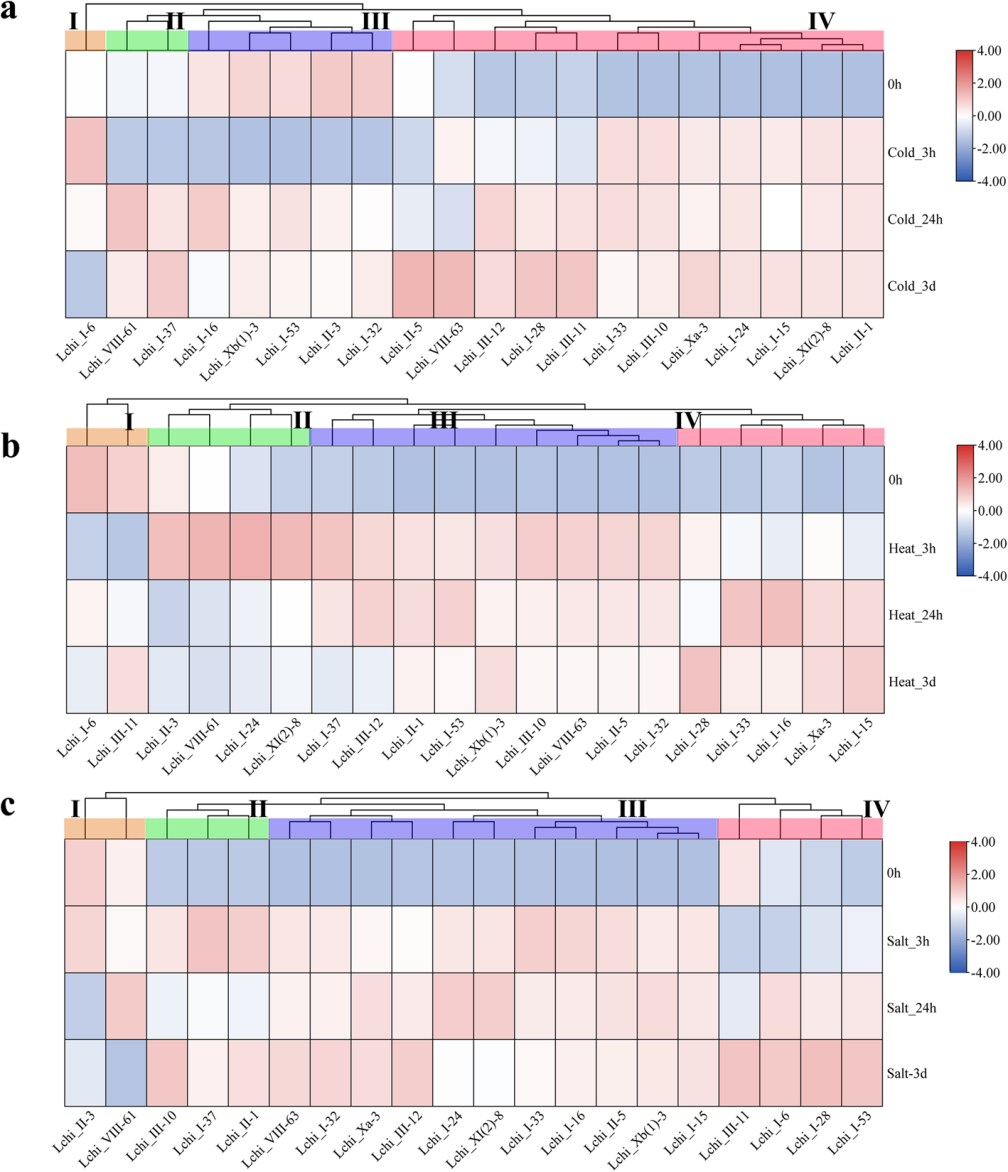
qPCR gene expression analysis of 20 *LchiLRR-RLK* genes in different abiotic stresses (cold, heat, and salt) at different time points (3 h, 24 h, and 3 d) generated using the TBtools software. Expression was analyzed based on the relative mRNA levels, which were reduced using the log value and maximized to 1. The final values were illustrated as heatmaps, with the key in the upper right corner. **a** cold, **b** heat, and **c** salt stress

To understand the possible responses of the *LchiLRR-RLK* genes to three abiotic stresses, twenty *LchiLRR-RLK* genes were selected for qPCR analysis based on the cis-regulatory results–that is *LchiLRR-RLK* genes with the highest representations of cis-regulatory elements responding to the abiotic stress. Additionally, their expression patterns in response to cold, heat, and salt stresses were analyzed over three time points, 3 h (h), 24 h, and 3 days (3d); and compared against the control (0 h) (Fig. 6). To thoroughly analyze the *LchiLRR-RLK* gene expression patterns, the genes were clustered into four expression pattern groups for all three abiotic stresses analyzed. Generally, the *LchiLRR-RLK* genes showed significant gene expression trends compared to the control (0 h). In cold stress (Fig. 6a), *LchiLRR-RLK*s exhibited a low to high expression trend; group one comprised one gene, *Lchi I-6*, which was upregulated 3 h after treatment onset and further downregulated until treatment termination. In group two, *Lchi_VIII-61* and *Lchi_I-37*, had a significant downregulation at 3 h and an upregulation at 24 h which was followed by a downregulation and upregulation at 3d for *Lchi_VIII-61* and *Lchi_I-37*, respectively. Group three comprised five genes, *Lchi_I-16*, *Lchi_Xb(1)-3*, *Lchi_I-53*, *Lchi_II-3*, and *Lchi_I-32*, which had significant downregulation at 3 h and an upregulation at 24 h and 3d which was insignificant compared to the 0 h. Group four had twelve genes significantly upregulated until treatment termination, except for *Lchi_III-11*, *Lchi_VIII-63*, and *Lchi_I-28* downregulated at 3 h. In addition, *Lchi_II-5* had low expression patterns at both the 3 h and 24 h time points. In total 60% of the LchiLRR-RLK genes showed significant upregulations during the cold stress especially at 24 h and 3d time points, suggesting that most of these genes respond to cold stress during the long time exposure to stress.

The heat stress was characterized by upregulations at different time points and alternating expression patterns till treatment termination (Fig. 6b). In detail, group one comprised two genes, *Lchi_I-6* and *Lchi_III-1* with insignificant expression patterns compared to the 0 h. The group two genes had an upregulation at 3 h only and were downregulated until treatment termination. Group three comprised nine *LchiLRR-RLK* genes which were highly upregulated till treatment termination, except for *Lchi_I-37* and *Lchi_III-12* which had a downregulation at 3d. In group four, genes showed an increasing expression pattern exhibiting peak upregulations at 3d except for *Lchi_I-33* and *Lchi_I-16* which had peak upregulation at 24 h followed by downregulation at 3d. Wholly, this result shows that *LchiLRR-RLK* genes respond to heat with an increasing expression pattern in proportion to time.

The salt stress gene expression patterns were characterized by upregulation trends (Fig. 6c). Noteworthy, almost all the genes had higher expression trends than the control. Group four consisted of four genes, *Lchi_III-11*, *Lchi_I-6*, *Lchi_I-28*, and *Lchi_I-53*. These genes showed the highest expression at 3d, and their expression trend was marked with an increasing upregulation from treatment onset; except for *Lchi_III-11* which had a significant downregulation at 3 h. The remaining groups had a similar expression pattern characterized by fairly upregulated trends throughout treatment. However, two genes, *Lchi_I-24* and *Lchi_XI(2)-8* were significantly downregulated at 3d, also group one members had a decreasing expression trend from treatment onset to termination.

## Discussion

The *LRR- RLK* genes constitute one of the largest gene groups in plants, playing a major role in plant growth and development, and biotic and abiotic responses [1]. Various *RLK* genes have been elucidated and their function demonstrated, including the *pathogenesis-related protein 5-like receptor kinase* (*PR5K*), *epidermal growth factor-like repeats* (*EGF*), *lectin-binding domain* (*LB*), *tumor necrosis factor receptor-like* (*TNFR*), and the *S-domain* [38]. On the other hand, the *L. chinense* like any other plant is susceptible to environmental cues threatening its survivability and productivity [25]. In this research, 233 LRR-RLK proteins were identified in the *L. chinense* genome carrying an extracellular domain (ECD), a transmembrane domain, and an intracellular kinase domain (KD) with the ECD branded by varying numbers of LRR repeats. Additional analysis revealed 15 LRR motifs with a 24 residue-long LRR domain, L/cxxLxxNxL/fsGxI/1PxxL/Ixx (Fig. 1), this was in agreement with the previous finding of a plant LRR denoted by a LxxLxxLxLxxNxLxGxIPxxLxx consensus sequence [39]. Investigations in the CDD and motif analyses also exhibited a conserved PK domain and an LRR domain. These findings demonstrate that the *LRR-RLK* genes are conserved in the *L. chinense* and may be involved in different plant growth and development functions, and biotic and abiotic responses. Adams et al. have shown that protein kinases are known for their vital functions in enzymatic roles due to the presence of their conserved subdomains [7]. Nonetheless, the *LRR-RLK* genes have been identified in several plant species, including *Populus trichocarpa,* citrus, Rosaceae, maize, and others. In this study, we identified 233 *LRR-RLK* genes, which were far more than in *Arabidopsis thaliana* (225) and less in *Oryza sativa* (332) in rice, this can be accounted for by the fact that *L. chinense* has a larger genome size of 1.749.3 Gb [40] compared to *A. thaliana* and *O. sativa* with 135 Mb and 430 Mb [41], respectively.

Additionally, inconsistencies in the gene family sizes can be related to gene duplication events. Research has related gene family expansion mainly due to two duplication events, tandem, and segmental duplication as sources of gene family expansion as it increases gene and genome densities [42]. This study showed that both the tandem and segmental duplications contributed 16% and 79% of the gene expansion of duplicated genes in the *L. chinense LRR-RLK gene* family. Similarly, previous studies in the Rosaceae gene groups have shown that tandem and segmental duplications are two major forms of gene family expansion contributing to almost 50% of the total gene family expansions [20]. In-depth analysis revealed that individual *LchiLRR-RLK* groups and subgroups expanded through tandem duplication. Interestingly in this research, the groups that expanded through the tandem duplications had the greatest numbers of the *LRR-RLK*s. Other research has also established that the expansion of the *LRR-RLK* gene family is enhanced due to their prime function in both development and defense responses, and continuous selection pressure imposed by the development complexities in the environment–reflecting *LRR-RLK* random gene drift [39]. Buttressing that *LRR-RLK*s in *L. chinense* are essential for development and environmental adaption–hence their huge protein family. Previous studies have also shown that the expansion of the *LRR-RLK* gene family has been contributed to through adaptive and non-adaptive evolution [28].

The origin of the *LRR-RLK* gene family remains a mystery although research has shown that the domain shuffling of the LRR and KD has led to the founding of the RLK subgroups [2]. To understand the phylogenetic relationships among the LRR-RLKs, we computed the phylogenetic tree using LRR-RLK full proteins from five plant species. The phylogenetic classification of the LRR-RLK proteins in *L. chinense* was similar to previous publications [34, 38]. The evolution of the LchiLRR-RLKs showed a divergence into several groups which emanated from an ancestral LchiLRR-RLK protein and we concluded that the LRR-RLK proteins evolved probably through duplication into several clades and groups that prompted specialization and function adaptation. In addition, the protein sequences clustered into 15 groups and 5 subgroups, renamed as I – XV (Fig. 3a and b), based on similar protein and domain arrangements. Groups XI (139) and VIII (104) had the most proteins, suggesting that protein duplication was relatively high in these groups. Based on tree topologies, we observed that the LchiLRR-RLKs were present in all the phylogenetic groups and even clustered with *A. thaliana* and other lower plants–this may entail that these genes are highly conserved and probably their expansion was during angiosperm WGD duplication events [43]. In addition, their presence may be related to group function specialization, for example, the PRK in subgroup II and PSY in subgroup XI were established in early plants due to their specific function in the pollen tube development [44–46]. In agreement with this finding, Liu et al. [1] have further published that subgroups I and VII-2 evolved from a common ancestor before the divergence of specific lineages and that most LRR-RLK subgroups were established in land plants before the divergence of moss [1]. In addition, the common presence of some LRR-RLKs in some phylogenetic groups of lower and higher plants such as *P. patens* and *L. chinense* also demonstrates the degree of protein conservativeness; since research has marked the mosses and lycophytes as early forms of plant life [47, 48]. Furthermore, the clustering of LchiLRR-RLKs from different plant species within the same groups such as *A. thaliana* and *L. chinense* may suggest that these proteins exhibit similar functional roles to their paralogues. In total, this finding shows that LchiLRR-RLKs are well conserved little function loss has been experienced due to gene mutations and related processes; and that they might possess central roles in the regulation of common developmental and defense pathways of different land plant lineages [43].

Plant LRR-RLKs are important membrane-localized receptors sensing various ligands to regulate plant developmental processes. Their diversity allows for response to several environmental stresses and actively functions in growth and developmental processes. For instance, the *somatic embryogenesis receptor kinase* (*SERK*), an *LRR-RLK* gene in wheat performs a dual function in heat tolerance and biotic stress resistance in *P. striiforms* F. *sp. triitcii* through interacting with the *TaDJA7* to activate the *HSP*s [16]. Previous research has also shown an *LRR-RLK* gene, *Phloem Intercalated with Xylem-Like 1* (*PXL1*) in Arabidopsis induced by cold and heat stress to phosphorylate *AtHIRD1* and *AtLHCA1* in regulating temperature fluctuations [17]. This research investigated the cis-regulatory elements in the promoter regions of identified *LRR-RLK* genes for predicting gene expression patterns and possible functional studies. Our results showed that the *LRR-RLK*s are actively involved in growth and development, and biotic and abiotic stress responses. In detail, the biotic response elements constituted a total of 55.22% of the total identified cis-elements. The identified cis-regulatory elements included the DRE-core, LTR, STRE, MYB, WRE3, and the WUN-motif. Typically, these regulatory elements encode the transcription of stress-responsive genes such as the *CBF*s (*C-repeat Binding Factors*) or *DREB*s (*Dehydration Responsive Elements*) [49], suggesting that *LchiLRR-RLK*s are invested in abiotic stress regulation. This result also led us to postulate that the *LRR-RLK* may interact with other stress-responsive genes in response to environmental stress [50]. A recent study in *Medicago truncatula* has shown that the *MtCTLK1* an *LRR-RLK* gene increased cold tolerance through inducing the expression of the *CBF*s and CBF-dependent cold responsive genes. Further research analysis indicates that *MtCTLK1* increases antioxidant enzyme activities and proline accumulation [14]. Providing possible insights that the *LchiLRR-RLKs* can also regulate the cold stress linking the CBF-cold response pathway [49].

To further unravel the possible functions of the identified *LchiLRR-RLKs* and their expression patterns in abiotic stresses, we used the qPCR expression analysis. We observed that a large proportion of the identified genes responded to both the temperature and salt stresses. Generally, the *LchiLRR-RLKs* were upregulated at different time points to varying extents and different groups showed differing expression trends. However, *Lchi_I-28*, *Lchi_Xa-3*, and *Lchi_I-15* had the highest expression patterns in all three stresses analyzed from treatment onset till termination. Implying that these genes may play a pivotal role in the regulation of these three stresses. In particular, a few *LchiLRR-RLKs* showed the highest upregulations in salt stress these included *Lchi_III-1*, *Lchi_I-6*, *Lchi_I-28*, and *Lchi_I-53* and the remaining *LchLRR-RLK* genes also respond fairly to salt stress. Therefore, we concluded that these members are highly involved in regulating salt stress. To support these findings, various *RLKs* from different subgroups, such as *RPK1*, *CYSTEINE-RICH RLK* (*CRK36*), *PROLINE-RICH-EXTENSIN-LIKE RLK4* (*PREK4*), and the *GUARD CELL HYDROGEN PEROXIDE-RESISTANT 1* (*GHR1*) in Arabidopsis have also been reported to regulate salt stress. However, little has been demonstrated on the mechanism of stress regulation [51–57]. In other studies, an LRR-RLK protein HSL3 was shown to negatively regulate stomatal closure by modulating the level of H_2_O_2_ in guard cells, thereby regulating drought and salt stress [13]. In Arabidopsis, an LRR-only protein belonging to group V was also demonstrated to regulate the abiotic stresses by interacting with the *DOF* (*DNA binding with One Finger*) and inducing its expression. Additional analysis also showed that the *lrr-op1* seeds with lower ABA levels were hypersensitive to abiotic stresses, implying that the *LRR-OP1* may also regulate the abiotic stress through the ABA-signaling pathways [58]. Another *LRR-RLK* member, *OSTLK* in rice was exhibited to regulate salt stress by regulating the ROS scavenging system, Na^+^/K^+^ ratio, and the MAPK signal pathways [59]. In tomato, an *MRK1* (*Multiple resistance-associated kinase1*) was significantly induced by the temperature stresses, additional studies showed increased transcript levels of the master regulators, the *C-repeat binding factor 1* (*CBF1*), and *Heat shock transcription TFs a-1a* (*HSFA1a*) [60]. Demonstrating that the *LRR-RLK* genes induce the expression of downstream genes thereby regulating the temperature stresses. Researchers in the *MRK1* gene have paraded it as a novel positive regulator of multiple stress and a potential breeding target for genetic engineering [60, 61].

This research shows that the *LRR-RLK* genes are conserved in the *L. chinense* and might have evolved from a single ancestor gene and diverged into different evolutionary groups for plant adaptation and functional responses in regulating environmental stresses including heat, cold, and salt.

## Conclusion

In this study, we have investigated the LRR-RLK transcription factors (TFs) in *L. chinense* and analyzed their structure arrangements, and expression patterns regulating cold, heat, and salt stresses. Through comprehensive bioinformatics analysis and experimental validations, we identified 233 *LchiLRR-RLK* genes localized on 17 chromosomes and 24 contigs. Analysis of their physiochemical properties through the protein motif numbers and arrangements, conserved domain, and gene structures exhibited that LRR-RLK proteins cluster together in different subgroups depending on similarity and conservation. Evolutionary studies demonstrated that these subgroups have a shared evolution history that indicates molecular function. A deeper survey into the *LchiLRR-RLK* genes promoter sequences evidenced that they carry cis-regulatory elements that respond to abiotic stresses including the low-temperature stress. Using the qPCR expression, we also concluded that a great number of *LchiLRR-RLK* genes may regulate heat, cold, and salt stress, especially members of subgroups VIII and III. Our findings demonstrate that the LchiLRR-RLK TFs serve as key regulatory nodes in the signaling pathways underlying abiotic stress responses–implying evidence that the *LchiLRR-RLKs* interact with downstream targeted genes for stress adaptation and tolerance. In addition, this study provides a fundamental base for understanding molecular mechanisms governing *LchiLRR-RLK*-mediated stress responses for plant improvements and sustainable agriculture. Moving forward, numerous avenues could be pursued further to research the *LchiLRR-RLKs*; such as their functional characterization, elucidating their precise roles in abiotic signaling pathways involving the generation of loss-of-function mutants, overexpression lines, and other physiological studies. Mechanistic insights into stress signaling will also unravel the downstream signaling components and molecular targets of the *LchiLRR-RLKs* using techniques such as chromatin immunoprecipitation and sequencing (CHIP-seq) and protein–protein interaction assays. Lastly, knowledge gained from this research can be used to genetically engineer stress-tolerant plants. This may involve the genetic manipulations of the *LchiLRR-RLKs* or their downstream target to enhance stress tolerance and improve plant productivity. In conclusion, progressive research of the *LchiLRR-RLKs* and their involvement in regulating abiotic stresses holds great promise in advancing our understanding of plant biology and developing novel strategies for plant enhancements in the face of global environmental challenges.

### Supplementary Information

Supplementary Material 1: Table S1. The primer sequences of LRR-RLK genes used in the qPCR analysis.

Supplementary Material 2: Table S2. The typical PKs and Tyr domains searched through the HMM program in TBtools.

Supplementary Material 3: Table S3. Physiochemical properties of LchiLRR-RLK proteins.

Supplementary Material 4: Table S4. The synonymous and nonsynonymous Ka/Kas Ratios of paralogous genes in the LchiLRR-RLK genes.

Supplementary Material 5: Table S5. The protein kinase and LRR domain positions within each LchiLRR-RLK protein. Different color schemes show respective domain position.

Supplementary Material 6: Table S6. Total numbers of the identified putative cis-elements in the promoter regions of *L. chinense *LRR-RLK genes, and their positions. Different colors show the total ranges of cis-elements present.

Supplementary Material 7: Fig S1. Shows 15 conserved motif logos present in the LRR-RLK gene family searched with the MEME suite online tool.

Supplementary Material 8: Fig S2. Motif structure and arrangement of LchiLRR-RLKs.

Supplementary Material 9: Fig S3. Phylogenetic analysis of the LRR-RLK protein sequences from 5 plant species.

Supplementary Material 10: Fig S4. The cis-regulatory elements present in the *L. chinense* promoter regions (1.5kb).

## Data Availability

Genome and gene model annotations files of *Liriodendron chinense* are available on the TreeGene database (https://treegenesdb.org/org/Liriodendron-chinense; accessed on 4 June 2023). Transcriptome datasets are also available on the NCBI website; the cold and heat stress accession number is PRJNA679089 (https://www.ncbi.nlm.nih.gov/bioproject/PRJNA679089, accessed on 4 June 2023). The drought stress accession number is PRJNA679101 (https://www.ncbi.nlm.nih.gov/bioproject/PRJNA679101/, accessed on 4 June 2023).

## Abbreviations

LRR-RLK: Leucine-rich repeat-like protein kinases
Lchi: *Liriodendron chinense*
ECD: Extracellular domain
KD: Intracellular kinase domain
*MtCTLK1-OE*: *M. truncatula *cold tolerance
LRR-RLK: Overexpressed
CBF: C-repeat-Binding Factor
PSKR: Phytosulfokine receptor
SERK: Somatic embryogenesis receptor kinase
PKs: Protein Kinases
HMM: Hidden Markov Models
CDD: Conserved domain
ML: Maximum likelihood
Ka/Ks: Synonymous and non-synonymous ratios
2^-ΔΔCt^: 2 (-delta delta CT)
DRE-core: Dehydration responsive elements-core
LTR: Low-temperature responsive elements
PPI: Protein-to-protein interaction
qPCR: Quantitative polymerase chain reaction
WGD: Whole genome duplication
TF: Transcription Factors
