# Supplementary figures and images for: Characterization, evolution, and abiotic stress responses of leucine-rich repeat receptor-like protein kinases (LRR-RLK) in Liriodendron chinense
Source: BMC Genomics. 2024 Jul 31;25:748. doi: 10.1186/s12864-024-10560-3 (PMC11292913; doi:10.1186/s12864-024-10560-3)

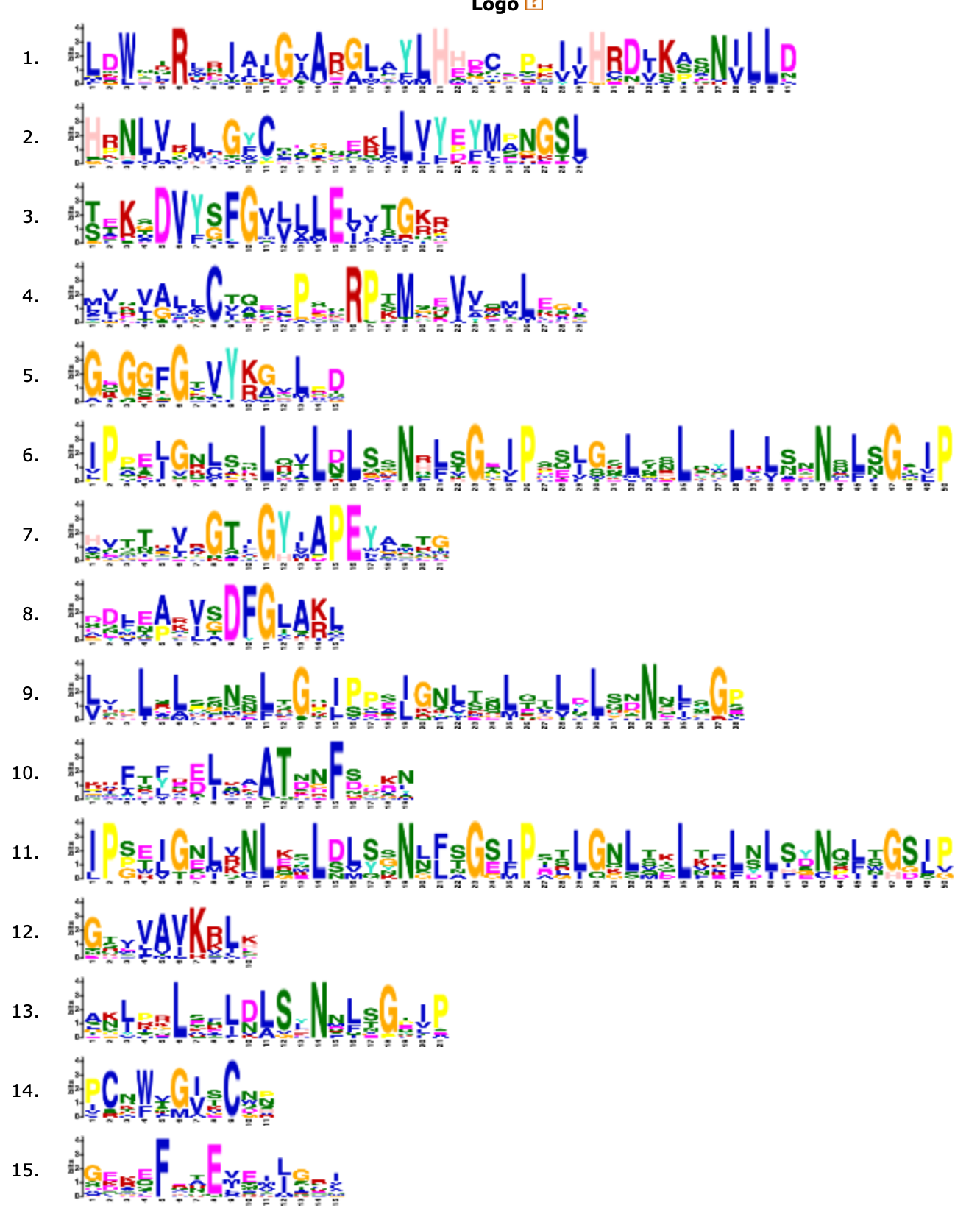

Supplement: Supplementary file 7 — Supplementary Material 7: Fig S1. Shows 15 conserved motif logos present in the LRR-RLK gene family searched with the MEME suite online tool. [file 12864_2024_10560_MOESM7_ESM.docx]

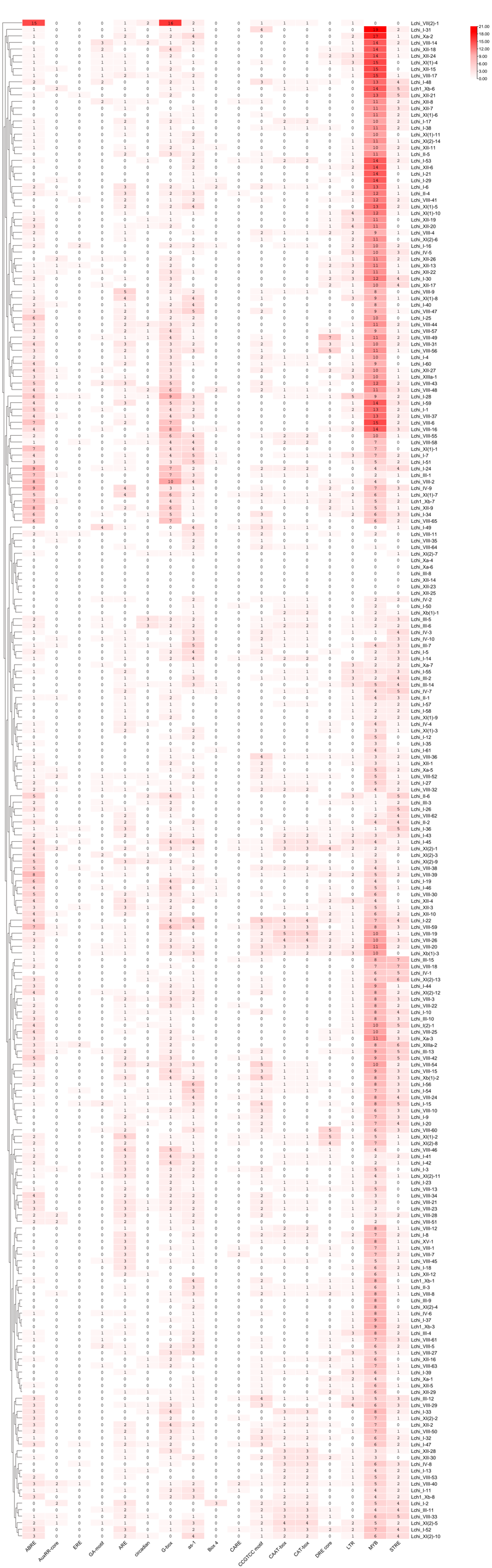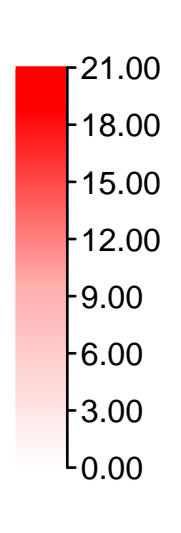

Supplement: Supplementary file 10 — Supplementary Material 10: Fig S4. The cis-regulatory elements present in the L. chinense promoter regions (1.5kb). [file 12864_2024_10560_MOESM10_ESM.pdf]
