# Supplementary material for: Characterization, evolution, and abiotic stress responses of leucine-rich repeat receptor-like protein kinases (LRR-RLK) in Liriodendron chinense
Source: BMC Genomics. 2024 Jul 31;25:748. doi: 10.1186/s12864-024-10560-3 (PMC11292913; doi:10.1186/s12864-024-10560-3)

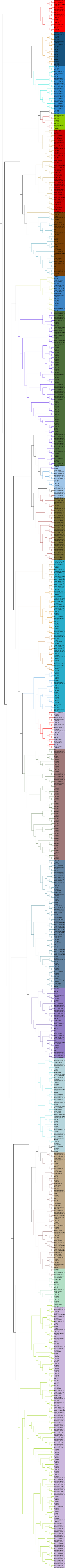

Supplement: Supplementary file 9 — Supplementary Material 9: Fig S3. Phylogenetic analysis of the LRR-RLK protein sequences from 5 plant species. [file 12864_2024_10560_MOESM9_ESM.pdf]
